# Reply to: A critical examination of a newly proposed interhemispheric teleconnection to Southwestern US winter precipitation

**DOI:** 10.1038/s41467-019-10531-3

**Published:** 2019-06-27

**Authors:** Antonios Mamalakis, Jin-Yi Yu, James T. Randerson, Amir AghaKouchak, Efi Foufoula-Georgiou

**Affiliations:** 1 Department of Civil and Environmental Engineering, University of California, Irvine, CA USA; 2 Department of Earth System Science, University of California, Irvine, CA USA

**Replying To** P. B. Gibson et al. *Nature Communications* https://doi.org/10.1038/s41467-019-10528-y (2019)

Gibson et al.^1^ comment on the physical mechanism suggested by Mamalakis et al.^2^ (hereafter referred to as M18), and question the first step of the newly proposed interhemispheric teleconnection, i.e., the atmospheric bridge, whereby sea surface temperature (SST) close to New Zealand (termed as NZI; the New Zealand Index^2^) modulates the SST in the Northwestern Pacific. Specifically, they suggest that there is no direct causal relationship between these key areas since the observed high statistical correlations between the corresponding SST anomalies can be largely explained by local SST memory and the El Niño-Southern Oscillation (ENSO), and that the increase in the correlations over the past four decades, as reported in M18, is likely the result of internal variability alone, not caused by historical forcings. Gibson et al.^1^ also argue that warm NZI is not associated with decreased convective activity and cloud cover over the east of the Philippines region, as suggested by M18. We appreciate the opportunity to debate these issues. In principle, we agree that a combination of more than one contributors can drive climate (and SST) variability in the northwestern Pacific (i.e., M18 did not argue that NZI is the only driver). However, we disagree with the general suggestion by Gibson et al.^1^ that the atmospheric bridge, as proposed in M18, is not supported by the data (observations and models). Here, we present evidence that indeed NZI carries non-redundant information that cannot be dismissed, it cannot be explained by internal variability alone, and we provide further analysis that supports the causal mechanism of the proposed atmospheric bridge. We also point out some intricate limitations in the analysis of Gibson et al., that might have affected their conclusions.

One of the arguments of Gibson et al.^1^ in challenging the NZI lagged association with northwestern Pacific SST is that the statistical correlations between these key areas decrease when accounting for (using partial correlation) local SST memory or ENSO (specifically, the Southern Oscillation Index - SOI). However, their analysis exhibits some limitations. Firstly, M18 did not argue that NZI is the only driver of SST variability in the northwestern Pacific. In fact, M18 already invoked the local SST memory in their proposed teleconnection mechanism (see step 2 in Figure 5 of M18). M18 simply argued for the emergence of a new western Pacific interhemispheric teleconnection, which in the ocean-atmosphere coupled system can affect, among other contributors, the north Pacific climate and ultimately the precipitation in the southwestern US (SWUS). Having clarified this, the meaningful question is not whether correlations of NZI and SST in the northwestern Pacific decrease when considering additional predictors/mechanisms (this is to be expected), but whether there are still patterns of statistically significant relations not explained by other predictors. As Gibson et al.’s^1^ own results suggest, a consistent pattern of statistically significant (local hypothesis testing at *a* = 0.05) correlations is still evident after accounting for both local SST memory and SOI (see their Fig. 1e, f), which means that NZI is not a redundant predictor of the SST in the northwestern Pacific. Thus, we do not think the results of Gibson et al.^1^ challenge our general suggestion. Note that although in the results of Gibson et al.^1^ (their Fig. 1f) there is an entire pattern of local statistically significant correlations, the authors seem to assess correlation significance solely by using the results from the false discovery rate (FDR) method. This can be misleading, because FDR only controls the likelihood of the type I error (rejecting a true null hypothesis), yet without controlling the likelihood of the type II error (not rejecting a false null hypothesis), which is known to increase as the number of multiple hypotheses increases (known as cost of multiplicity control^3^).

**Fig. 1:**
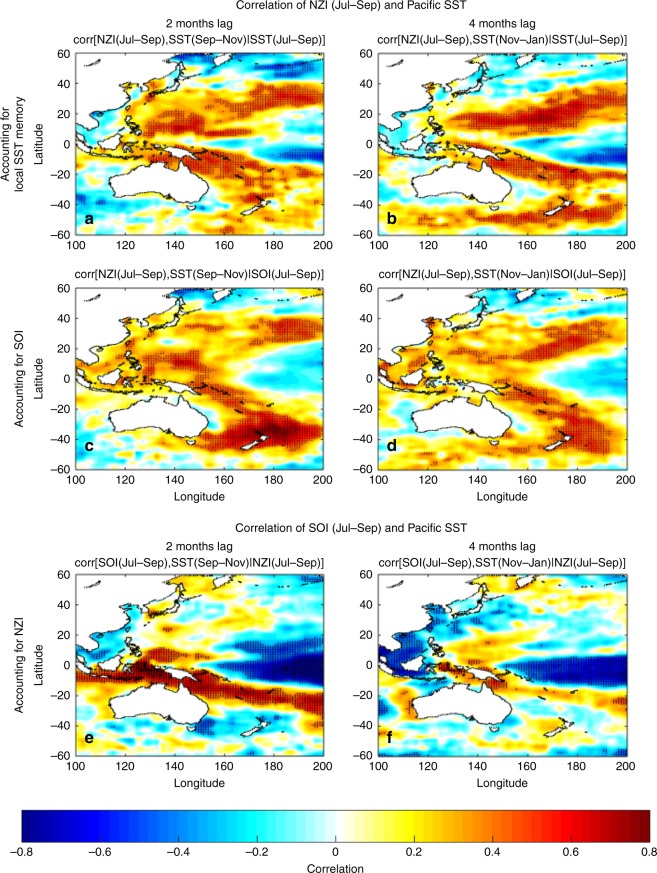
NZI interhemispheric connection when accounting for local SST memory and ENSO in 1982–2015. **a** Correlation map of NZI during Jul–Sep and Pacific SST during Sep–Nov, after accounting for the SST memory in each grid point. Before calculating correlations all series were detrended. Black dots indicate statistical significance at *a* = 0.05. **b** Same as in **a**, but using SST during Nov–Jan. **c**, **d** Same as in **a**–**b**, but after accounting for the Southern Oscillation Index (SOI). **e**, **f** Same as in **c**–**d**, but SOI is correlated with Pacific SST, after accounting for the NZI. It is evident that NZI correlations with SST in the northwestern Pacific remain high and statistically significant even after accounting for local SST memory and/or SOI. In contrast, when accounting for NZI, correlations of SOI with SST in the northwestern Pacific are not statistically significant

Moreover, assessing the solidity of the atmospheric bridge mechanism by using only a small box (20° × 10°) to capture the SST variability in the northwestern Pacific can be misleading, especially when there is no investigation of how sensitive the general conclusions are to the choice of that box. The specific box east of the Philippines was tentatively used in our original study to summarize and compare the western Pacific dynamics before and after 1980s (Figure 7 in M18); a very different context than the present investigation of causality and mechanism assessment. Thus, in order to rigorously assess the arguments of Gibson et al.^1^, we investigate herein the effect of local SST memory and SOI on the correlation between the late summer NZI and lagged SSTs over the entire western Pacific (not only over a specified box). We find that statistically significant correlation patterns are evident in the northwestern Pacific (with correlations reaching 0.7 in specific areas, see Fig. 1), even after accounting for local SST memory (Fig. 1a, b) or SOI (Fig. 1c, d). When reproducing Fig. 1 of Gibson et al.^1^ by considering a larger region in the northwestern Pacific (of comparable size to the NZI region and defined as the 30° × 15° box of 130°E–160°E and 5°N–20°N – we call the average SST in that box as the East of the Philippines Index, EPI), we find that NZI-EPI correlations remain high and statistically significant at *a* = 0.05, after accounting for the EPI memory (see Supplementary Fig. 1b). Particularly in the summer period, correlations on the order of 0.6 are still evident, which means that NZI leads EPI SST in the following winter. Accordingly, our results show that when accounting for SOI, strong NZI-EPI correlation patterns are still evident (see Supplementary Fig. 1c). All above results support that the lagged association between late summer-early fall NZI and northwestern Pacific SST is robust, and is not undermined by SOI or local SST persistence. Lastly, we note that if there was no direct causal relationship between NZI and EPI, and SOI was the driver of this interhemispheric connection (as implied by Gibson et al.^1^), then following the reasoning by Gibson et al.^1^, one would expect that the correlation of SOI with the northwestern Pacific SSTs would be significant even after accounting for the NZI variability. However, our results indicate that this is not the case (not shown in Gibson et al.^1^). Particularly, when accounting for the NZI variability, significant correlations of SOI and Pacific SST appear only in the tropics and do not extend to the northern Pacific (Fig. 1e, f). Moreover, statistically significant SOI-EPI correlations are only found after November (see Supplementary Fig. 1f), which suggests that in late summer, NZI carries non-redundant information for the fall-winter SST over the northwestern Pacific. In summary, although SOI and local SST memory are known to account for a fraction of SST variability in the northwestern Pacific, our results show that they do not undermine the robustness of the NZI-EPI statistical relationship.

The second point raised by Gibson et al.^1^ concerns the decadal changes in the NZI-EPI relationship. Gibson et al.^1^ use SST data from the Community Earth System Model-Large ENSemble project (CESM-LENS, see ref. ^4^) and show that there are some ensemble members (realizations) where NZI-EPI are highly coupled and some where they are not statistically related. Based on this result, they cast doubt on the statement by M18 that the recent strengthening in the NZI-EPI relationship may be attributed, among others, to climate change. Specifically, Gibson et al.^1^ conclude that “any apparent lagged correlations found between these regions are not likely a consistent or emerging feature of the climate system under historical forgings, but instead occasionally arise due to stochastic internal climate variability”. We respectfully disagree with this statement, since their results suggest that internal variability can indeed contribute in shaping the NZI-EPI relation (something that M18 did not argue against) but they cannot exclude historical forcings from being a contributor as well. To more extensively explore this, here we use the same set of LENS simulations that Gibson et al.^1^ use and compare the simulated NZI-EPI correlations before and after 1970s. Our results show that indeed there is a strengthening in the NZI-EPI relationship after the 1970s (see Fig. 2), which is in accordance with the findings by M18. The latter is indicated not only by the increase of the ensemble mean correlations between NZI and EPI (especially in the late-summer season) but also by the decrease of the inter-ensemble variability in the recent decades, which indicates convergence of the ensembles. Thus, in accordance with M18, the LENS simulations suggest that the increased NZI-EPI correlations can be an emerging feature of the climate system under historical forcings, and cannot be solely explained by internal variability. Note that if stochastic internal variability was the only driver of the changes in the observed NZI-EPI correlations, then no changes should be apparent in the LENS ensemble mean correlations between the two periods, since changes in individual realizations would not be synchronized, and would cancel each other out.

**Fig. 2:**
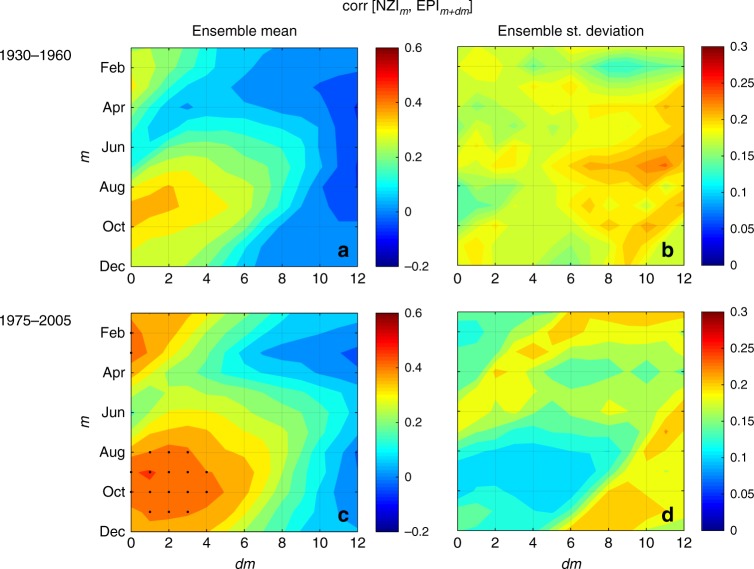
Changes in the strength of correlation between the NZI and EPI regions derived from the CESM-LENS project. Ensemble mean **a**, **c** and ensemble standard deviation **b**, **d** of time-lagged correlations between NZI (averaged over the month indicated in the vertical axis) and EPI (lagged forward in time as indicated in the horizontal axis) in 1930–1960 **a**, **b** and in 1975–2005 **c**, **d**. Before calculating correlations all series were detrended. Black dots indicate statistical significance at *a* = 0.05. A higher level of agreement among ensembles and a clear increase in the NZI-EPI correlations is observed after 1975

The last claim by Gibson et al.^1^ is that warm NZI is not associated with decreased convective activity and cloud cover over the east of the Philippines region, as suggested by M18. Here, we show that this is not the case by performing composite and correlation analyses with multiple reanalysis and satellite datasets. When we consider the composite differences in the Jul–Nov SST, omega velocity, total cloud cover (TCC), and incoming solar radiation (ISR), between the five warmest and coldest NZI years, the mechanism suggested by M18 is supported (Fig. 3). In late boreal summer, the intertropical convergence zone migrates close to the Philippines (see ref. ^5^) and the southern Hadley cell expands the most^6^, dominating the atmospheric meridional circulation over the western tropical Pacific (see e.g., ref. ^7^ and Supplementary Figure 7 in M18) and connecting the areas of NZI and EPI. In warm NZI years, both the descending motions over the southwestern Pacific (close to New Zealand) and ascending motions over the northwestern Pacific (close to the Philippines) weaken in all pressure levels, which translates to a weakened Hadley circulation (not reversed as Gibson et al.^1^ imply) relatively to the cool NZI years (see the north-south dipole in Fig. 3b). Accordingly, the suppressed cloud formation over EPI results in increased solar radiation reaching the surface (see Fig. 3c, d), which can induce positive SST anomalies in this region (process 1 in Figure 5 of M18). In the Nov–Mar period, the cyclonic activity southwestward of the Philippines helps maintain the SST anomalies in the northwestern and central Pacific (process 2 in Figure 5 of M18), while a persistent high pressure ridge is evident off the coast of California, deflecting the jet stream to the north (process 3 in Figure 5 of M18) and introducing positive precipitation anomalies over the northwestern US and negative precipitation anomalies (drought) over the SWUS. Similar conclusions are obtained using correlation analysis and multiple other atmospheric variables (see Supplementary Figs. 2 and 3 of the present study). Particularly, a clear north-south dipole pattern is obtained over the western Pacific, which reveals the modulation effect of the NZI on the regional Hadley circulation. Finally, we note that the different findings of Gibson et al.^1^ regarding the effect of NZI on the convective activity over the northwestern Pacific might be attributed to the following reasons. First, Gibson et al.^1^ base their conclusions on the ERA-Interim dataset which, as shown in Supplementary Fig. 3 of the present study, exhibits the lowest level of agreement with other reanalysis and satellite datasets in reproducing the relationship of NZI and the convective activity over EPI. Second, their results presented in Fig. 2c, f, i are based on only 17 years of climatology (compared to our analysis based on 34 years, 1982–2015), which is not a sufficient size for statistical (correlation or composite) analysis.

**Fig. 3:**
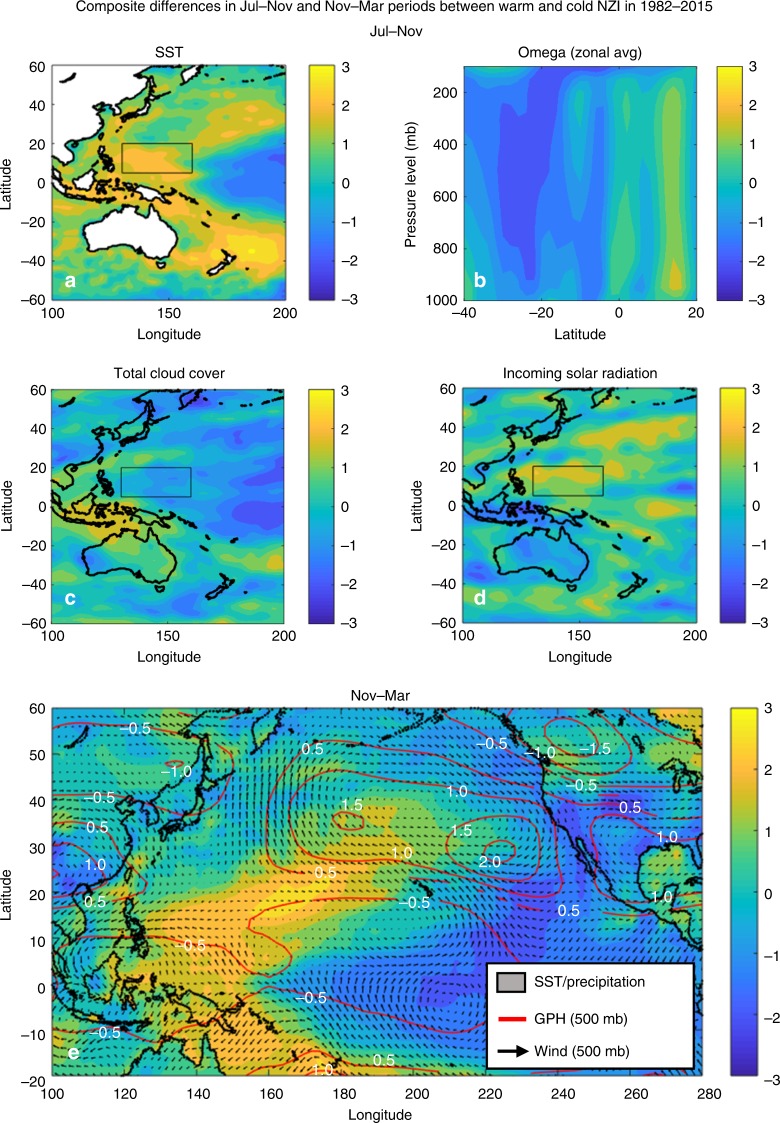
Composite differences between 5 warmest and 5 coldest NZI years during 1982–2015. **a** Jul–Nov SST, **b** Jul–Nov zonal average omega velocity over 100°E–200°E (positive sign corresponds to decreased ascending motion), **c** Jul–Nov total cloud cover (TCC), **d** Jul–Nov incoming solar radiation (ISR) at the surface, and **e** Nov–Mar SST (shading over ocean), land precipitation (shading over land), geopotential height (GPH; contours) and horizontal wind (vectors) at 500 mb pressure level. All values have been standardized by dividing with the standard deviation of the respective series in 1982-2015. In **a**, **c**, **d**, the EPI box is also shown. As suggested in M18, results support that warm NZI is associated with decreased convective activity and cloud cover, and increased incoming solar radiation over the east of the Philippines

In conclusion, we thank Gibson et al.^1^ for their critical analysis and exploration of the proposed physical mechanism of the NZI teleconnection, and for providing us with the opportunity to further examine and strengthen our original arguments. We believe their letter and our response advance the important topic of understanding sources of predictability of regional precipitation and their physical underpinnings in the western Pacific. As noted already by M18, given the short observational record that is available, whether NZI SST anomalies alone are sufficient to modulate the meridional circulation over the western Pacific demands extensive further testing, which will require the performance and analysis of a hierarchy of well-designed and targeted numerical simulations. We hope that this undertaking will be the scope of future work in our group and others in the community.

## Supplementary information

Supplementary Information

## Data Availability

All data used in this study are freely available. Time series of SOI (monthly scale) were obtained from NOOA website: https://www.esrl.noaa.gov/psd/data/climateindices/list. In this study, the Optimum Interpolation SST dataset was used, which is freely available at https://www.esrl.noaa.gov/psd/data/gridded/data.noaa.oisst.v2.html, while omega velocity, OLR, ISR and TCC were obtained from 20^th^ century reanalysis project https://www.esrl.noaa.gov/psd/data/gridded/data.20thC_ReanV2c.html. CESM-LENS^4^ data were obtained from http://www.cesm.ucar.edu/projects/community-projects/LENS/data-sets.html. OLR and TCC products in Supplementary Fig. 3 are from the NCEP-DOE reanalysis and the ERA-Interim reanalysis, and were downloaded from https://www.esrl.noaa.gov/psd/data/gridded/data.ncep.reanalysis2.gaussian.html and http://apps.ecmwf.int/datasets/data/interim-full-daily/levtype=sfc, respectively, while satellite precipitation is from PERSIANN-CDR dataset (chrsdata.eng.uci.edu). Also, upon reasonable request, the data and code that support the findings of this study can be provided by the corresponding author.
