# Supplementary material for: Reply to: A critical examination of a newly proposed interhemispheric teleconnection to Southwestern US winter precipitation
Source: Nat Commun. 2019 Jun 27;10:2918. doi: 10.1038/s41467-019-10531-3 (PMC6597566; doi:10.1038/s41467-019-10531-3)
Supplement: Supplementary file 1 — Supplementary Information [file 41467_2019_10531_MOESM1_ESM.pdf]

Supplementary Information

Reply to: A critical examination of a newly proposed interhemispheric teleconnection to Southwestern US winter precipitation

by Mamalakis et al.

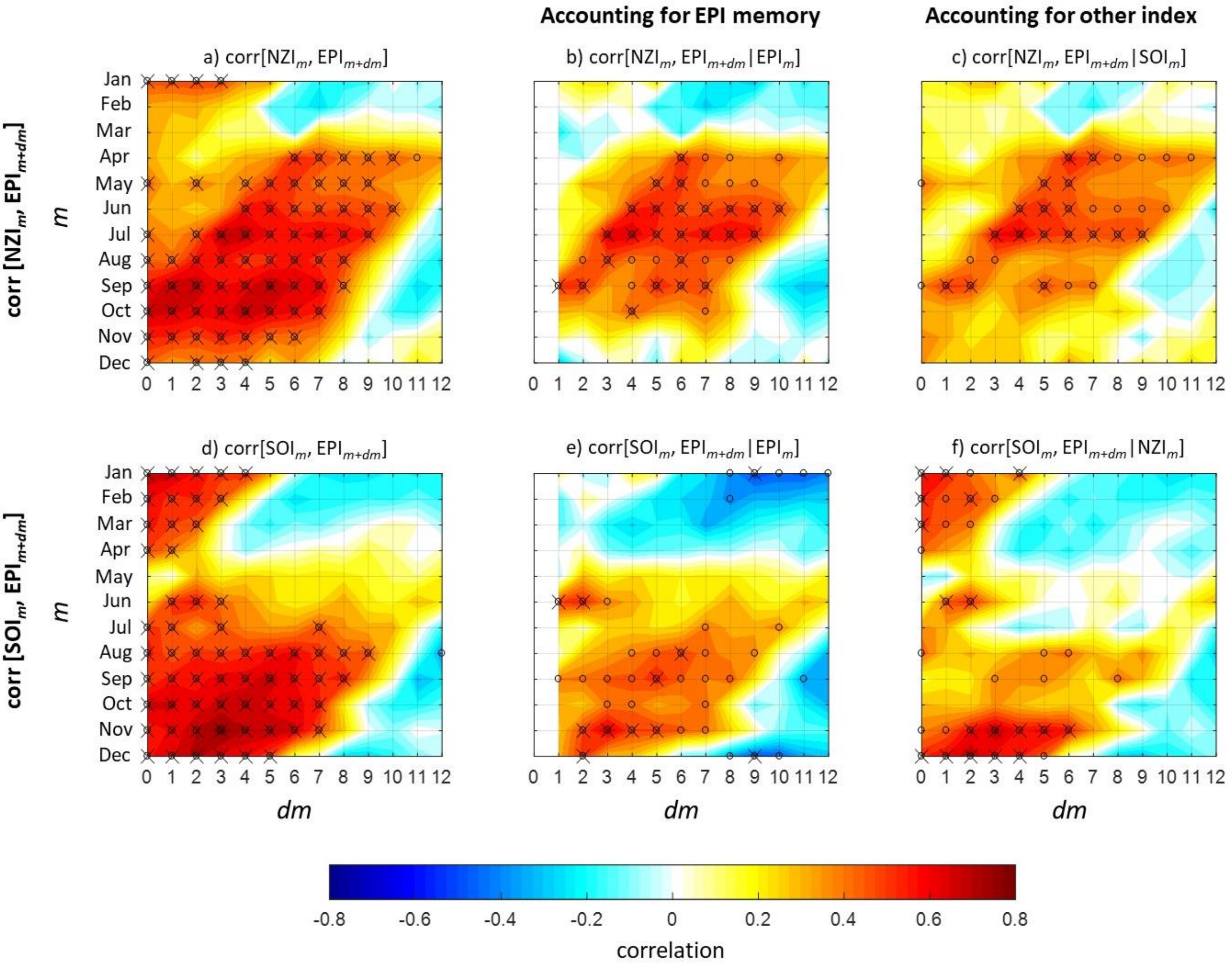

Supplementary Figure 1: *Upper Panels:* a) Time-lagged correlations between NZI (averaged over the month indicated in the vertical axis) and SST in the region east of the Philippines (EPI) (box in 5–20°N and 130–160°E; lagged forward in time as indicated in the horizontal axis) in 1982–2015. Before calculating correlations all series were detrended. b) Same as in (a), but when the memory (monthly persistence) of EPI is taken into account. c) Same as in (a), but when the effect of SOI is taken into account. Black circles indicate statistical significance at a = 0.05, while black crosses indicate global significance at a_{global} = 0.05. For the latter, we have used the false discovery rate as in Gibson et al., with $a_{\mathrm{FDR}} = 2a_{\mathrm{global}} = 0.1$, to account for dependence of the local tests, as suggested by Wilks (2016). *Bottom Panels:* Same as in upper panels, but using (d) SOI as predictor instead of NZI, and accounting for (e) EPI memory and (f) NZI variability. The coherent patterns of statistically significant NZI-EPI correlations when accounting for EPI memory and SOI establish the interhemispheric connection on its own right and not slaved to local SST memory or ENSO.

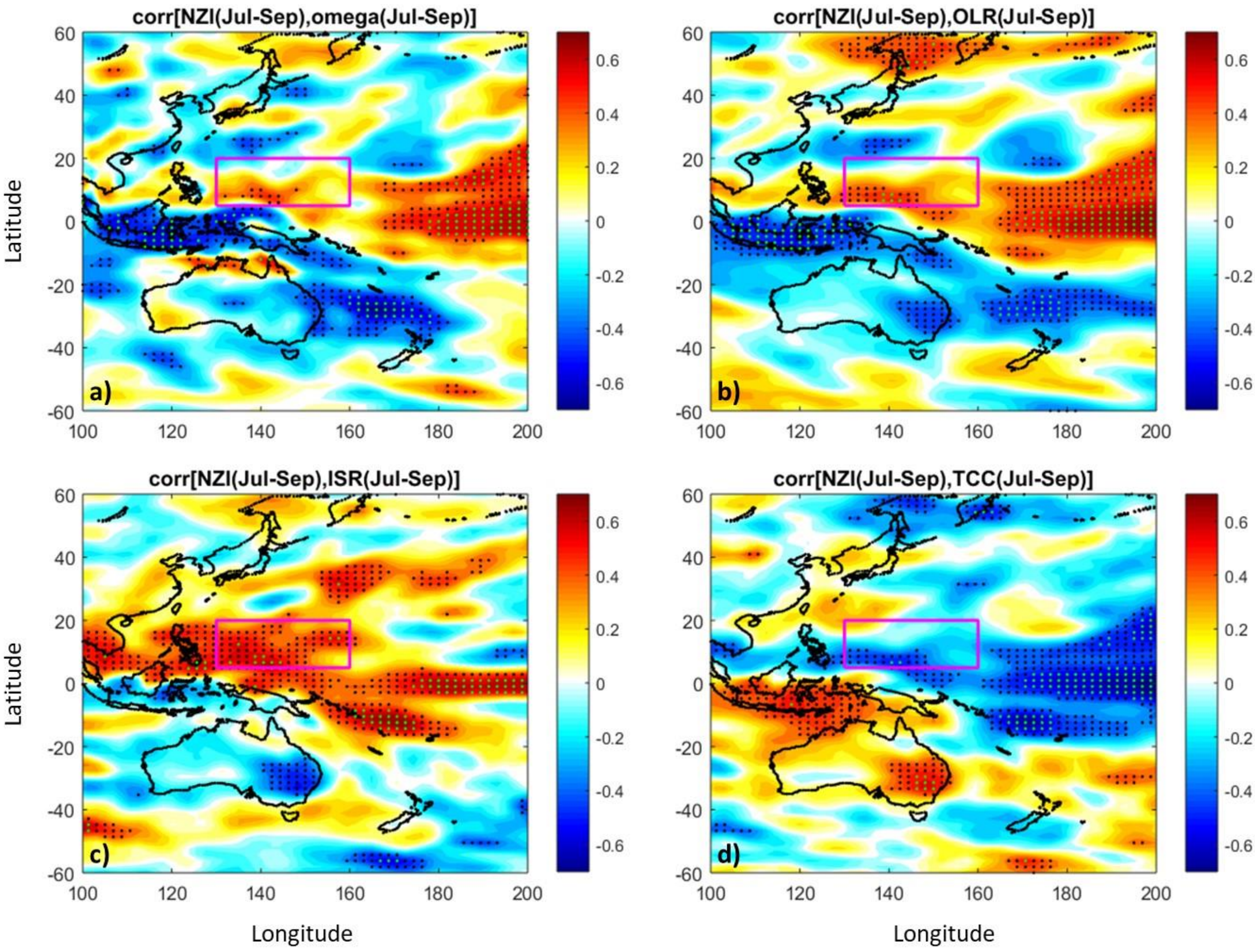

Supplementary Figure 2: Jul-Sep correlation maps (1982-2014) between NZI and a) omega velocity at 500mb (positive sign corresponds to descending or decreased ascending motion), b) outgoing longwave radiation (OLR) at the top of the atmosphere, c) incoming solar radiation (ISR) at the surface, and d) total cloud cover (TCC). Before calculating correlations all series were detrended. Black dots indicate statistical significance at $a = 0.05$, while green dots indicate global significance at $a_{\mathrm{global}} = 0.05$. For the latter, we have used the false discovery rate as in Gibson et al., with $a_{\mathrm{FDR}} = 2a_{\mathrm{global}} = 0.1$, to account for dependence of the local tests, as suggested by Wilks (2016). EPI region is indicated with a magenta box. NZI is calculated using the Optimum Interpolation SST, while other series are obtained from the 20th Century Reanalysis project. All results show that positive NZI is associated with decreased convective activity and increased ISR over the northwestern Pacific. Note also that a clear north-south dipole pattern is obtained over the western Pacific, which reveals the modulation effect of the NZI on the regional Hadley circulation.

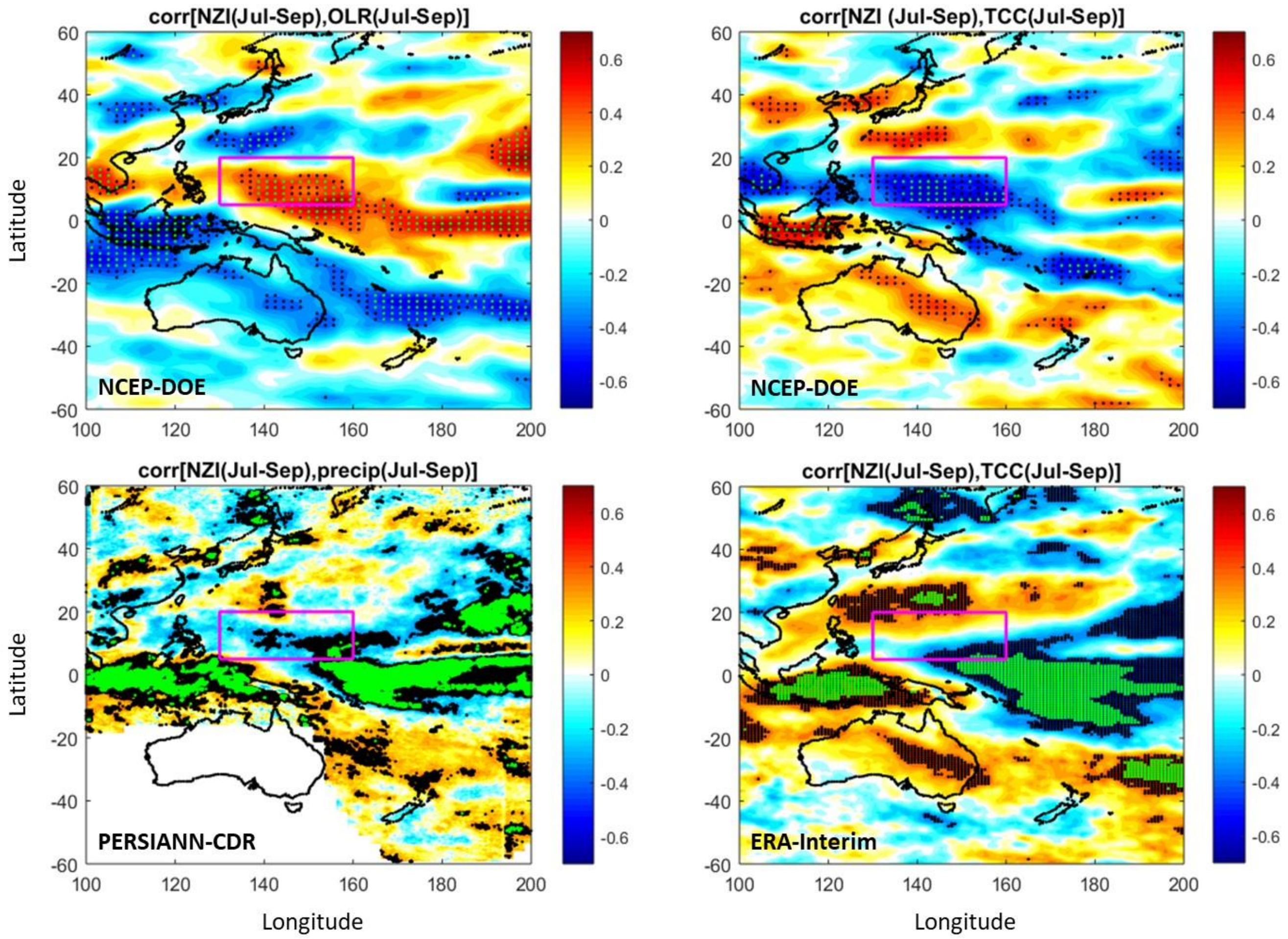

Supplementary Figure 3: Same as in Supplementary Figure 2, but TCC and OLR are obtained from the NCEP-DOE dataset in the top plots, while satellite precipitation is obtained from PERSIANN-CDR, and TCC is from the ERA-Interim project in the bottom plots. Relatively to results presented in other subplots and in Supplementary Figure 2, results from the ERA-Interim project (used in Gibson et al.) are unclear as to the relationship between NZI and the convective activity over the EPI.

Reference

Wilks, D. S. (2016) "The stippling shows statistically significant grid points": How research results are routinely overstated and overinterpreted, and what to do about it. *Bulletin of the American Meteorological Society*, **97**(12), 2263-2273.
